# Strong Lattice Softening Induced by Atomic Mismatch in Meta‐Phase Thermoelectrics

**DOI:** 10.1002/advs.202514105

**Published:** 2025-10-06

**Authors:** Kunpeng Zhao, Min Li, Hexige Wuliji, Haotian Gao, Hongyi Chen, Pengfei Qiu, Zhengyang Zhou, Xun Shi

**Affiliations:** ^1^ State Key Laboratory of Metal Matrix Composites School of Materials Science and Engineering Shanghai Jiao Tong University Shanghai 200240 China; ^2^ State Key Laboratory of High Performance Ceramics and Superfine Microstructure Shanghai Institute of Ceramics Chinese Academy of Sciences Shanghai 200050 China; ^3^ University of Chinese Academy of Sciences Beijing 100049 China; ^4^ School of Materials Science and Engineering and Institute of Materials Genome & Big Data Harbin Institute of Technology Shenzhen 518055 China; ^5^ College of Chemistry and Chemical Engineering Central South University Changsha Hunan 410083 China

**Keywords:** argyrodite, atomic mismatch, lattice softening, lattice thermal conductivity, thermoelectric

## Abstract

Conventional strategies for suppressing lattice thermal conductivity *κ*_L_ typically focus on maximizing phonon scattering to reduce phonon mean free path. Such reductions, however, are limited to the interatomic spacing or phonon wavelength. Alternatively, herein, an effective approach is proposed to lower phonon velocity by introducing atoms with significant atomic mismatch into the crystal lattice of three meta‐phases. Specifically, substituting Te for S in Ag_8_SnS_6_ and Cu_2_S, or Sn for Si in Mg_2_Si considerably increases the atomic mass and weakens the chemical bonding, causing notable reductions in the sound velocity. This reduction further leads to an amorphous‐like, extremely low lattice thermal conductivity *κ*_L_ across the whole temperature range. Consequently, we achieve outstanding thermoelectric performance in these atomic mismatched meta‐phases, with a maximum *zT* of 1.0 for Ag_8_SnS_4.99_Te, 1.1 for Mg_2_Si_0.5_Sn_0.5_, and 2.0 for Cu_2_S_0.5_Te_0.5_. The work demonstrates a new approach to manipulating thermal conductions through lattice softening, providing a promising pathway for designing high‐performance thermoelectric materials.

## Graphical abstract

This work demonstrates the validity and universality of atomic mismatch‐induced lattice softening across three metaphase systems: Ag_8_Sn(S,Te), Cu_2_(S,Te) and Mg_2_(Si,Sn).

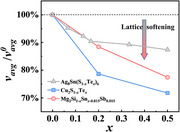

## 1 Introduction

Thermoelectric (TE) technology, which facilitates the direct conversion of heat into electricity and vice versa, has garnered significant attention for its potential to address global energy challenges and mitigate environmental issues.^[^ ^1^, ^2^, ^3^, ^4^ ^]^ The energy conversion efficiency of TE materials is quantified by the dimensionless TE figure of merit, *zT* = *α* ^2^ *σT*/*κ*, where *α*, *σ*, *κ*, *T* are the Seebeck coefficient, electrical conductivity, total thermal conductivity, and absolute temperature, respectively. Enhancing TE performance faces substantial challenges due to the intricate interdependence of the electrical and thermal properties, all of which are influenced by carrier concentration and scattering mechanisms. In terms of electrical properties, various strategies such as band convergence,^[^ ^5^, ^6^ ^]^ band broadening,^[^ ^7^ ^]^ resonant doping,^[^ ^8^, ^9^ ^]^ and disorder‐induced electronic localization^[^ ^10^, ^11^ ^]^ have been proven effective in enhancing the power factor *α* ^2^ *σ*. On the thermal side, suppressing lattice thermal conductivity (*κ_L_*), the only independently tunable material parameter, is the most commonly used approach. Most existing strategies focus on minimizing the phonon relaxation time (*τ*) through methods like all‐scale hierarchical structuring,^[^ ^12^ ^]^ the introduction of liquid‐like ions,^[^ ^13^, ^14^ ^]^ entropy engineering,^[^ ^15^, ^16^, ^17^ ^]^ structural modularization,^[^ ^10^ ^]^ or anharmonic lattice dynamics.^[^ ^18^, ^19^, ^20^ ^]^

The lattice thermal conductivity of a material is not only related to the phonon relaxation time *τ* but is also highly sensitive to the material's sound velocity (*v_s_*).^[^ ^21^, ^22^, ^23^, ^24^ ^]^ This sensitivity can be illustrated through a spectral analysis of phonon thermal conductivity:^[^ ^21^, ^25^, ^26^ ^]^

(1) $$\kappa_{L}=\frac{1}{3}\int_{0}^{\omega_{max}}C_{s}\tau v_{g}^{2}d\omega$$

where *C_s_* is the spectral heat capacity, and *v_g_* is the group velocity. In an ideal uniform elastic medium, *v_g_* and *v_s_* are similar in the long‐wavelength limit. Therefore, manipulating the sound velocity *v_s_* (i.e., lattice stiffness) in a material is a powerful means of controlling *κ_L_*. Han et al.^[^ ^24^ ^]^ revealed that aliovalent doping can introduce additional charge carriers and enhance screening effects, which consequently soften and slow down optical phonons. Slade et al.^[^ ^23^ ^]^ concluded that an increase in carrier concentration promotes lattice softening and thereby reduces lattice thermal conductivity. In fact, for materials with complex structures or at high temperatures, when the phonon relaxation time is already very low, reducing *v_s_* by softening the crystal lattice is expected to have a more significant impact on *κ_L_* than phonon scattering.

Theoretically, the *v_s_* of a material is primarily related to the atomic mass *M* and the chemical bond strength *F* between atoms,^[^ ^27^, ^28^ ^]^ i.e., $v_{s}\propto \sqrt{\frac{F}{M}}$. To achieve a reduction in *v_s_*, it is necessary to decrease the chemical bond strength and increase the atomic mass. This can be accomplished by incorporating a high concentration of solute atoms with a large atomic mismatch into the lattice. However, when there is a significant difference in atomic size, atomic mass, and electronegativity between the solute and host atoms, considerable lattice stress and material instability can occur. Thus, the solubility of the mismatched atoms is generally low,^[^ ^29^, ^30^ ^]^ which in turn limits the applicability of this method for lattice softening.

Our previous study has demonstrated that materials with significant atomic size mismatch can be stabilized by introducing adaptable sublattices with large diffusion coefficient or large atomic displacement parameter.^[^ ^31^, ^32^, ^33^ ^]^ Specifically, the adaptable ions rapidly migrate to suitable positions, altering the coordination surroundings of the mismatched anions and consequently alleviating substantial stress. The interplay between adaptable and mismatched ions leads to the formation of meta‐phases,^[^ ^31^, ^32^, ^33^ ^]^ where the mismatched anions form an ordered crystalline sublattice and the adaptable cations constitute a disordered sublattice. Herein, we selected three meta‐phase systems, i.e., Ag_8_Sn(S,Te)_6_, Cu_2_(S,Te) and Mg_2_(Si,Sn), to manipulate the atomic structure and lattice stiffness. Despite the large mismatch in atomic radius, atomic mass, and electronegativity between Te and S or between Si and Sn, we successfully obtain single‐phase structures over a broad compositional range for all three material systems. Remarkably, the introduction of mismatched atoms in these compounds substantially reduces the sound velocity, yielding amorphous‐like extremely low lattice thermal conductivity and superior TE performances.

## 2 Results and Discussion

### 2.1 Structural Evolution of Ag_8_Sn(S_1‐_ *_x_* Te*_x_*)_6_

**Figure 1:**
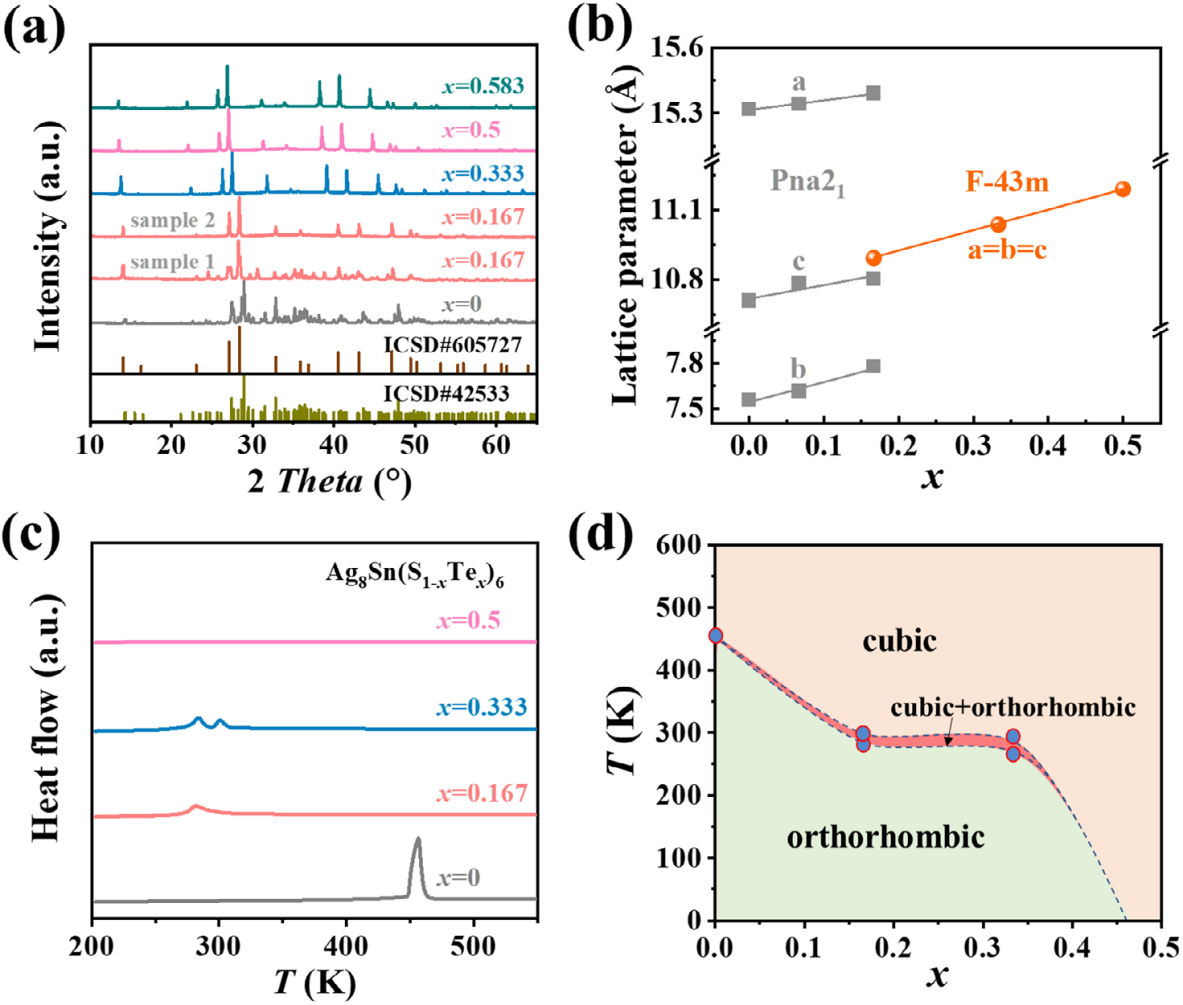
Structural characterization and phase transition. a) Room temperature powder X‐ray diffraction (PXRD) patterns for Ag_8_Sn(S_1‐_ *_x_* Te*_x_*)_6_ (*x* = 0, 0.167, 0.333, 0.5, 0.583). b) Lattice parameters as a function of alloying content *x*. c) Temperature dependence of heat flow. d) Pseudo phase diagram of Ag_8_Sn(S_1‐_ *_x_* Te*_x_*)_6_ against the composition and temperature.

Given that the three meta‐phase systems share many similarities in structures and properties, we first focus on Ag_8_Sn(S,Te)_6_ for in‐depth discussion, and then extend our study to Cu_2_(S,Te) and Mg_2_(Si,Sn). We synthesized a series of Ag_8_Sn(S_1‐_ *_x_* Te*_x_*)_6_ (*x* = 0, 0.067, 0.133, 0.167, 0.333, 0.5, 0.583) and S‐deficient Ag_8_SnS_5‐_ *_y_* Te (*y* = 0.01, 0.02, 0.03) samples using the high‐temperature melting method followed by hot pressing. The energy‐dispersive spectroscopy (EDS) results indicate that the distribution of all elements is uniform in samples with *x* ≤ 0.5, and the measured composition is very close to the nominal one (Figure S1, Supporting Information). This suggests that the solubility of Te in Ag_8_SnS_6_ can reach up to 50%, despite the significant atomic mismatch between Te and S (Table S1, Supporting Information). The measured powder X‐ray diffraction (PXRD) patterns demonstrate a composition‐driven structural transition from orthorhombic to cubic phase at room temperature after alloying Te in Ag_8_SnS_6_ (**Figure** **1**a; Figure S2, Supporting Information). With the increase of Te content, the refined lattice parameters increase linearly within the orthorhombic or cubic phase region (Figure 1b), confirming the successful alloying of Te at S sites. Interestingly, the sample with *x* = 0.167 could exhibit both the orthorhombic and cubic phases at room temperature, indicating that a phase transition might occur near room temperature for *x* = 0.167, which was verified by our differential scanning calorimetry (DSC) results. As shown in Figure 1c, the DSC curve of *x* = 0 sample exhibits a sharp exothermic peak at 456 K, indicating a temperature‐driven phase transition from the orthorhombic to cubic phase at this temperature point. The high‐temperature XRD measurements also verify the structural evolution with temperature (Figure S3, Supporting Information). When the Te content increases to *x* = 0.167, the exothermic peak decreased to ≈290 K, and the intensity of the peak was significantly reduced, suggesting a lower energy barrier for the phase transition. This also well explains why we could obtain both the orthorhombic and cubic phases at room temperature. When the Te content increases to *x* = 0.333, 2 weak adjacent peaks appear in the DSC curve, suggesting that this composition does not undergo a direct orthorhombic‐to‐cubic phase transition. Variable‐temperature XRD measurements further support this conclusion: the *x* = 0.333 sample shows a pure orthorhombic phase at 250 K and transforms into a single cubic phase at 350 K (Figure S4, Supporting Information). However, due to the narrow temperature interval between the two DSC peaks, we currently lack high‐quality XRD data within this intermediate range. According to the rule of phase boundary continuity, a two‐phase region must exist between the two single‐phase regions. When the Te content further increases to *x* = 0.5, the exothermic peak completely disappears, indicating no phase transitions across the measured temperature range. Based on the results of PXRD and DSC, we draw a pseudo phase diagram of Ag_8_Sn(S_1‐_ *_x_* Te*_x_*)_6_ against the composition and temperature, as shown in Figure 1d. Through this phase diagram, we can more intuitively see the regions occupied by the orthorhombic and cubic phases, thus enabling better control of the crystal phase structure by adjusting the composition or temperature.

**Figure 2:**
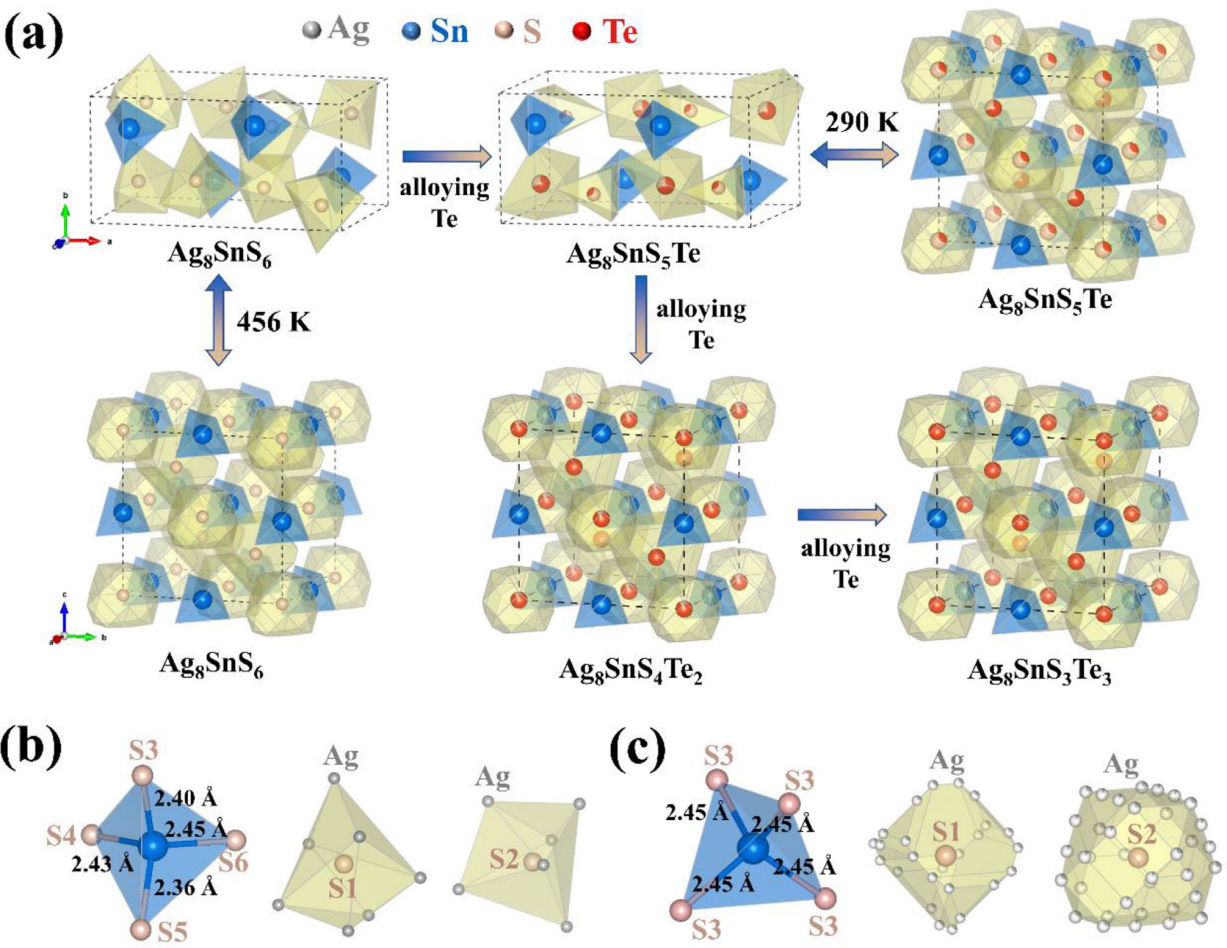
Visualization of the orthorhombic and cubic structures. a) Structural evolution of Ag_8_Sn(S_1‐_ *_x_* Te*_x_*)_6_ at different temperatures and/or compositions. b) Coordination environments of Sn and S atoms in the orthorhombic structures. c) Coordination environments of Sn and S atoms in the cubic structures. The atomic site occupancy of Ag is indicated by partial coloring of the atoms.

**Figure 3:**
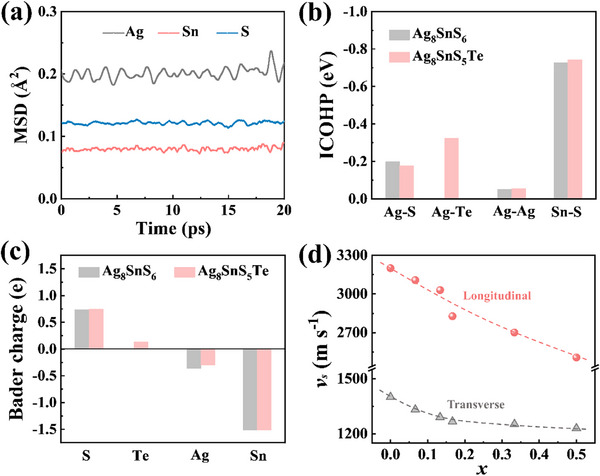
Chemical bonding and lattice softening. a) Mean square displacement (MSD) of Ag, Sn, and S in Ag_8_SnS_6_ obtained from molecular dynamics (MD) simulation at 300 K. b) Integrated crystal orbital Hamilton population (ICOHP) for the chemical bonds in Ag_8_SnS_6_ and Ag_8_SnS_5_Te. c) Bader charge for the different elements in Ag_8_SnS_6_ and Ag_8_SnS_5_Te. d) Measured sound velocities as a function of alloying content. The dashed lines are guides to the eyes.

To gain a clearer understanding of the atomic occupancy, we determined the crystal structures of Ag_8_Sn(S_1‐_ *_x_* Te*_x_*)_6_ through Rietveld refinements of PXRD data. The Rietveld refinement results are shown in Figure S5 (Supporting Information). The obtained crystallographic information and refined parameters are listed in Tables S2–S6 (Supporting Information). Both the low‐temperature orthorhombic structure and high‐temperature cubic structure of Ag_8_SnS_6_ are composed of [SnS_4_] tetrahedra and two different [SAg*_x_*] polyhedra that are interwoven and stacked (**Figure** **2**a; Figure S6, Supporting Information). However, in the orthorhombic structure, each Sn atom is coordinated with four different S atoms (S3, S4, S5, and S6) with varying Sn−S bond lengths, resulting in a distorted [SnS_4_] tetrahedron (Figure 2b). In contrast, in the cubic structure, the Sn atom is coordinated with four identical S atoms (S3) with the same Sn─S bond length, forming a regular [SnS_4_] tetrahedron (Figure 2c). The orderly distributed Ag atoms in the orthorhombic structure and the disorderedly distributed Ag atoms in the cubic structure are both primarily coordinated with S1 or S2, forming weak Ag─S bonds and constituting complex [SAg*_x_*] polyhedra. Due to the large number of Ag lattice sites, the Ag─S bond lengths vary over a wide range, from 2.3 to 2.9 Å, demonstrating high adaptability and flexibility. Consequently, in both structures, the mismatched Te preferentially occupy the S1 and S2 sites bonded exclusively with Ag. The radial distribution function (RDF) analysis (Figure S7, Supporting Information) reveals that the average Ag─Te bond length is ≈2.76 Å, much longer than that of the Ag─S bond (2.49 Å). The broader RDF peaks for Ag─X and Ag─Ag compared to Sn─S suggest greater variability in the interatomic distances and a higher degree of disorder of Ag. Structural refinement and molecular dynamics simulations further elucidate the disorder and dynamic nature of Ag atoms. As shown in Tables S2–S6 (Supporting Information) and **Figure** **3**a, Ag exhibits large values of atomic displacement parameters (ADP) and mean square displacement (MSD), enabling them to migrate rapidly to suitable lattice sites and form optimal chemical bonds with the mismatched atoms, thereby releasing lattice stress and stabilizing the structure.^[^ ^31^, ^33^ ^]^

### 2.2 Chemical Bonding and Lattice Softening in Ag_8_Sn(S_1‐x_Te_x_)_6_

The structural adaptability and flexibility arise primarily from the weak Ag─S or Ag─Te bonds. As shown in Figure 3b, the average integrated crystal orbital Hamilton population (ICOHP) value of the Ag─S bond is only 0.2 eV, significantly lower than the 0.8 eV for Sn─Te bond, indicating a weak covalent character in the Ag─S bond. Moreover, the Bader charge analysis shows that in Ag_8_SnS_6_, each S atom receives ≈0.74 electrons, while each Sn and Ag atom loses 1.51 and 0.36 electrons, respectively. The relatively low Bader charge, i.e., low charge transfer, implies that the ionic component of the Ag─S bonds is also very weak. Given the high content of weakly bonded Ag ions, the overall chemical bonds in Ag_8_SnS_6_ are very weak. As a result, the sound velocities in Ag_8_SnS_6_ are very low, with shear and longitudinal sound velocities being only 1400 and 3200 m s^−1^, respectively, which are much lower than those of classic TE materials such as Bi_2_Te_3_ and PbTe.^[^ ^22^, ^34^ ^]^ The Debye temperature, derived from the sound velocities, is only 140 K, while the Grüneisen parameter is remarkably high at 2.4. These characteristics can be traced back to the weak atomic bonding and a high degree of anharmonicity in its lattice vibrations.

Upon alloying Te at S sites, the chemical bonding characteristics of Ag─X bonds undergo noticeable changes. On one hand, the ICOHP value of Ag─Te bond increases to 0.32 eV, indicating a slight enhancement in the covalent character of Ag─Te bond relative to the Ag─S bond. On the other hand, the Bader charge value of the alloyed Te atoms (only 0.13) is much lower than that of S (0.74, see Figure 3c) since Te is much less electronegative than S. Combining the much longer bond length of Ag─Te compared to Ag─S, we believe that the overall Ag─X chemical bonds in Ag_8_Sn(S_1‐_ *_x_* Te*_x_*)_6_ are significantly weakened. This weakening of the chemical bonds, coupled with the increased atomic mass introduced by Te, give rise to a marked decrease in the material's sound velocity. Specifically, for the sample with *x* = 0.5, the shear and longitudinal sound velocities are merely 1230 and 2509 m s^−1^, respectively. The averaged sound velocity is 1381 m s^−1^, representing a reduction of 13% compared to Ag_8_SnS_6_ (1581 m s^−1^). Ten repeated measurements of the sound velocity of the same sample show a standard deviation of less than 1%.

Overall, the lattice softening comes from the weakening of bonding strength (*F*) and the increase of atomic mass (*M*). For simple lattices in the long‐wavelength limit of acoustic phonons, one may approximate $v_{s}\propto \sqrt{\frac{F}{M}}$. Under such an idealized scenario, it would be possible to roughly separate the contributions from bond weakening and increased atomic mass to the observed lattice softening. However, in the present Ag_8_Sn(S,Te)_6_ system, the situation is far more complicated: multiple types of bonds coexist (Ag─S, Sn─S, and even Ag─Ag), with mixed covalent and ionic characteristics. Furthermore, Te substitution modifies both the mass and the bonding environment in a nontrivial and coupled manner. As such, it is not feasible to cleanly disentangle the relative contributions of mass increase and bond weakening in this system.

**Figure 4:**
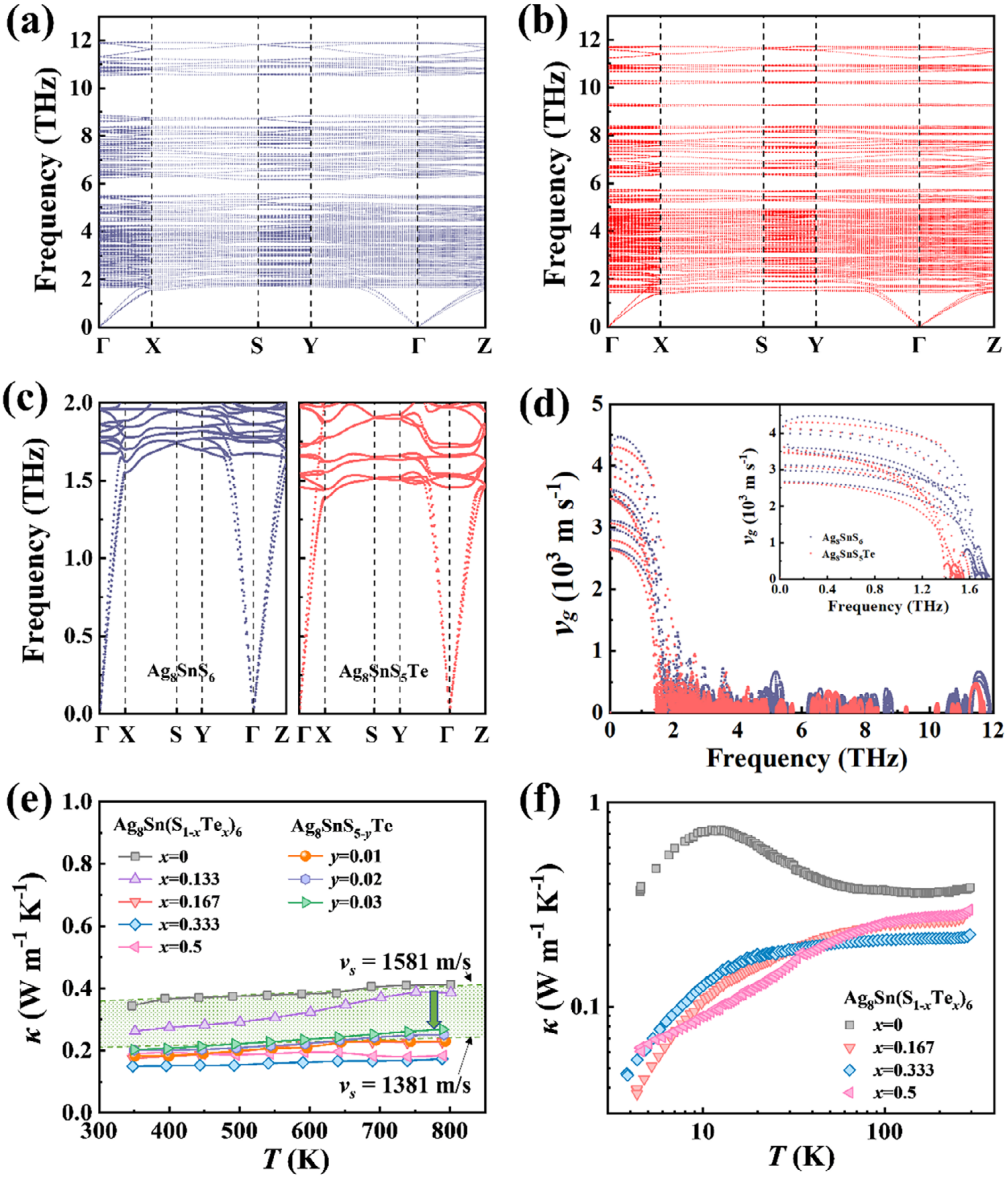
Thermal transport properties. Calculated phonon dispersion relations for a) Ag_8_SnS_6_ and b) Ag_8_SnS_5_Te. c) Comparison of the acoustic phonon dispersions between Ag_8_SnS_6_ and Ag_8_SnS_5_Te. d) Group velocity versus phonon frequency calculated from the phonon dispersion relations. The inset highlights the group velocity of acoustic phonons. e) Temperature dependence of total thermal conductivity *κ* for Ag_8_Sn(S_1‐_ *_x_* Te*_x_*)_6_ and Ag_8_SnS_5‐_ *_y_* Te above 300 K. The upper and lower boundaries of the shadow area are calculated using Equation (1) with the same parameter *A*_0_ but different sound velocities. f) Thermal conductivity below 300 K.

Further calculations of the phonon spectra of Ag_8_SnS_6_ and Ag_8_SnS_5_Te were performed to verify the lattice softening phenomenon. As shown in **Figure** **4**a–c, the phonon spectra exhibit no imaginary frequencies, indicating the structural stability of the metaphase compounds. Due to the large primitive cell and weak chemical bonds, Ag_8_SnS_6_ exhibits a high number of optical branches and a low cutoff frequency *ω_m,_*_ac_ of acoustic phonons (1.7 THz). Within the acoustic phonon range (0–1.7 THz), the group velocities calculated from the phonon dispersions are approximately between 2500 m s^−1^ and 4500 m s^−1^ (Figure 4d), and they decrease sharply as the frequency approaches *ω_m,_*_ac_. By contrast, across the entire optical phonon range (1.7–12 THz), the group velocities remain below 700 m s^−1^. These results indicate that acoustic phonons play the dominant role in heat transport. Upon Te alloying, the cutoff frequency *ω_m,_*_ac_ of acoustic phonons further decreases to 1.4 THz, resulting in lower group velocities for Ag_8_SnS_5_Te compared to Ag_8_SnS_6_ at the same frequency (inset in Figure 4d). This trend is in good agreement with the observations from our experiments. In addition, both the group velocities and cutoff frequency (*ω_m,_*_op_) of the high‐frequency optical phonons in Ag_8_SnS_5_Te are reduced relative to pristine Ag_8_SnS_6_. This clearly demonstrates that Te incorporation softens not only the acoustic phonons but also the optical phonons.

### 2.3 Thermal Transports of Ag_8_Sn(S_1‐x_Te_x_)_6_

The extremely low sound velocities induced by lattice softening in Ag_8_Sn(S_1‐_ *_x_* Te*_x_*)_6_ lead to an expectation of exceptionally low lattice thermal conductivity. Figure 4c illustrates the temperature‐dependent total thermal conductivity (*κ*) for Ag_8_Sn(S_1‐_ *_x_* Te*_x_*)_6_ above 300 K. Given the relatively low electrical conductivity, the electronic contribution to thermal conductivity in Ag_8_Sn(S_1‐_ *_x_* Te*_x_*)_6_ is almost negligible. Therefore, the total thermal conductivity is primarily contributed by the lattice thermal conductivity *κ*_L_. The observed *κ* and *κ*_L_ of pristine Ag_8_SnS_6_ are only ≈0.4 W m^−1^ K^−1^, a value close to the theoretical glass‐limit calculated from Cahill's model (0.43 W m^−1^ K^−1^). The extremely low *κ*_L_ of Ag_8_SnS_6_ originates from multiple factors, including the disordered Ag sublattice, weak chemical bonding, large unit cell, strong lattice anharmonicity, and the possible presence of low‐lying multi‐Einstein oscillators. We calculated the impact of point defect scattering on *κ*_L_ using the Callaway model and found that in materials with such low *κ*_L_, the contribution of point defect scattering to the reduction of *κ*_L_ is very weak above 300 K. This is understandable because in Ag_8_SnS_6_ the phonon scattering has been maximized and the phonon mean free path has approached the interatomic distance. However, unlike scattering phonons, reducing the group velocity of phonons holds promise for further reducing the lattice thermal conductivity, as demonstrated by our Ag_8_Sn(S_1‐_ *_x_* Te*_x_*)_6_ metaphase. As shown in Figure 4c, the lattice thermal conductivity of Ag_8_Sn(S_1‐_ *_x_* Te*_x_*)_6_ is merely 0.15–0.26 W m^−1^ K^−1^, which is one of the lowest among known thermoelectrics and only two‐thirds to half that of the Ag_8_SnS_6_ matrix. According to spectral Callaway model:^[^ ^21^, ^25^, ^26^ ^]^

(2) $$\kappa_{L}=\frac{{\left(6\pi^{2}\right)}^{2/3}\bar{M}}{V^{2/3}4\pi^{2}\gamma^{2}T}\;\langle v_{g}^{3}\rangle =A_{0}\;v_{s}^{3}$$

the *κ*_L_ is proportional to the cube of sound velocity above the Debye temperature. A decrease of 13% in sound velocity would result in a 34% reduction in the *κ*_L_. In other words, the reduction of *κ*_L_ in Ag_8_Sn(S_1‐_ *_x_* Te*_x_*)_6_ can largely be attributed to the lattice softening effects.

We also measured the low‐temperature (4−300 K) thermal conductivity of Ag_8_Sn(S_1‐_ *_x_* Te*_x_*)_6_ using the physical property measurement system (PPMS), with the results shown in Figure 4d. It can be observed that at low temperatures, especially below 50 K, the difference in *κ* (also *κ*_L_) between Ag_8_Sn(S_1‐_ *_x_* Te*_x_*)_6_ and Ag_8_SnS_6_ is more pronounced. Specifically, the *κ*_L_ of *x* = 0.5 sample at 10 K is 0.09 W m^−1^ K^−1^, which is only one‐eighth of that of Ag_8_SnS_6_ (0.72 W m^−1^ K^−1^). Moreover, Ag_8_Sn(S_1‐_ *_x_* Te*_x_*)_6_ and Ag_8_SnS_6_ also exhibit distinctly different temperature dependencies. The *κ_L_* of Ag_8_SnS_6_ shows a pronounced Umklapp peak at ≈10 K, indicative of the typical thermal transport behavior of crystalline solids.^[^ ^35^ ^]^ In contrast, the *κ*_L_ of Ag_8_Sn(S_1‐_ *_x_* Te*_x_*)_6_ monotonically increases throughout the entire temperature range without a peak, resembling the behavior of amorphous materials.^[^ ^36^ ^]^ Unlike the strong phonon scattering at high temperatures, the reduction of *κ*_L_ at low temperatures (below 50 K) is attributed not only to the influence of sound velocity, but also to the extra phonon scattering by point defects, structural disorder, and low‐lying multi‐Einstein oscillators.^[^ ^37^ ^]^

### 2.4 Electrical Transports and *zT* Values of Ag_8_Sn(S_1‐x_Te_x_)_6_

**Figure 5:**
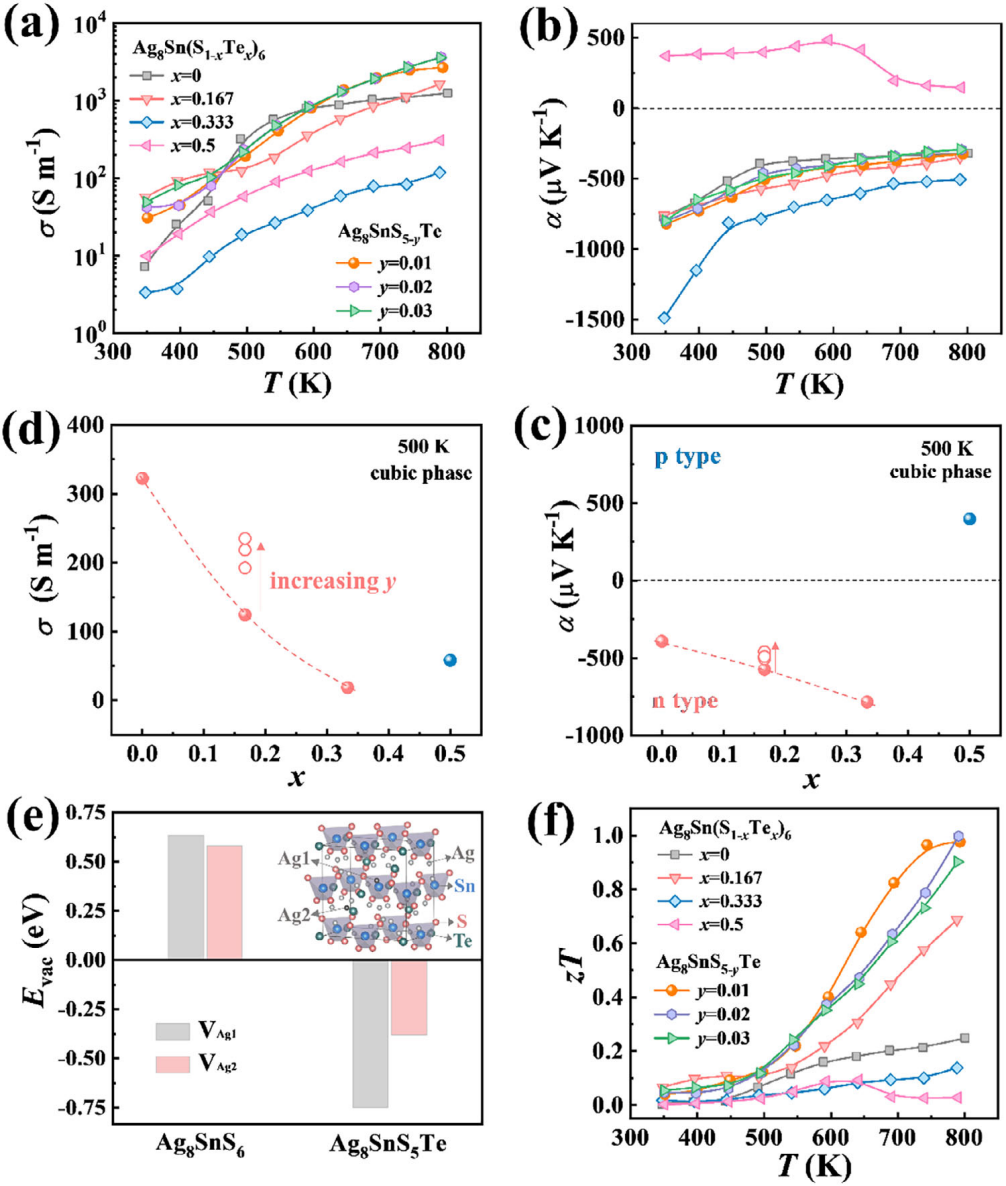
Electrical transport properties and *zT* values. Temperature dependence of a) electrical conductivity *σ* and b) Seebeck coefficient *α* for Ag_8_Sn(S_1‐_ *_x_* Te*_x_*)_6_ and Ag_8_SnS_5‐_ *_y_* Te. c) electrical conductivity *σ* and d) Seebeck coefficient *α* as a function of alloying content *x*. at 500 K at which all the samples are cubic phases. e) Calculated formation energy *E*_vac_ of two types of Ag vacancies in Ag_8_SnS_6_ and Ag_8_SnS_5_Te. f) Temperature dependence of TE figure of merit *zT*.

**Figure** **5** shows the electrical transport properties for Te alloyed Ag_8_Sn(S_1‐_ *_x_* Te*_x_*)_6_ and S‐deficient Ag_8_SnS_5‐_ *_y_* Te. Pristine Ag_8_SnS_6_ exhibits an electrical conductivity *σ* of merely 7 S m^−1^ at room temperature, which monotonically increases to 1249 S m^−1^ at 800 K, demonstrating semiconducting behavior (Figure 5a). Ag_8_SnS_6_ shows different temperature dependencies below 450 K and above 500 K, which is attributed to the order‐to‐disorder phase transitions in the material. Opposing to the trend of *σ*, the Seebeck coefficient *α* of Ag_8_SnS_6_ decreases from 769 µV K^−1^ at room temperature to 320 µV K^−1^ at 800 K (Figure 5b). The negative *α* values indicate that electrons are the dominant charge carriers in pristine Ag_8_SnS_6_. Upon Te alloying, notable changes are observed in both *σ* and *α*. When *x* ≤ 0.333, the *σ* gradually decreases with increasing Te content, while the absolute value of *α* gradually increases (Figure 5c,d). When the Te content reaches *x* = 0.5, the *α* changes from negative to positive, indicating a shift in the dominant carrier type from electrons to holes.

Such electrical transport behavior can be well explained by the data of defect calculations (Figure 5e). The electrical transports of A^+^_12‐y_B^y+^C^2−^_6_ argyrodites are mainly controlled by A defects:^[^ ^38^, ^39^, ^40^ ^]^ when A is in excess, A interstitials (donors) exist in the lattice, resulting in n‐type conductivity; whereas when Ag is deficient, A vacancies (acceptors) are present, leading to p‐type conductivity. Therefore, we calculated the formation energies *E*_vac_ of two types of Ag vacancies in Ag_8_SnS_6_ and Ag_8_SnS_5_Te. For Ag_8_SnS_6_, the *E*_vac_ is ≈0.6 eV (Figure 5e), indicating that Ag is relatively difficult to be removed from the lattice, thus exhibiting n‐type conductivity. In Ag_8_SnS_5_Te, the *E*_vac_ becomes negative, with −0.75 eV for *V*_Ag1_ and −0.3 eV for *V*_Ag2_, implying that Ag vacancies will spontaneously form. It should be noted that the calculated vacancy formation energies are obtained at 0 K and for neutral vacancies only, without considering temperature effects or possible charged states of the defects. In addition, the precise calculation of defect formation energies is known to be challenging, and large errors can occur depending on the choice of exchange‐correlation functional, supercell size, and reference chemical potentials. Therefore, while the exact value of *E*_vac_ may carry uncertainty, the calculations reliably capture the trend that Ag vacancies are favored upon alloying Te in Ag_8_SnS_6_. Consequently, with increasing Te content, the electron concentration in the material gradually decreases, while the hole concentration gradually increases, and ultimately shifts to hole‐dominated conduction at a Te content of *x* = 0.5. The change in *E*_vac_ may be related to the alteration of Ag─X bonds. After alloying Te at S sites, the overall Ag─X bonds are weakened, making Ag more likely to break free and leave the lattice sites. Since Te incorporation decreases the electron concentration in Ag_8_Sn(S_1‐_ *_x_* Te*_x_*)_6_, the impact of charge‐carrier‐driven lattice softening, as reported by Slade et al.,^[^ ^23^ ^]^ can be excluded from the present system. In addition to alloying, adjusting the content of anions can also regulate the electrical transport properties. As shown in Figure 5, by reducing the S content, the *σ* increases significantly while the *α* decreases, implying the increase of electron concentrations.

Combining the measured *S*, *σ* and *κ*, we calculated the power factor (*PF*) and figure of merit *zT* as a function of temperature. Owing to its low electrical conductivity, pristine Ag_8_SnS_6_ exhibits a relatively *PF*, reaching only 1.2 µW cm^−1^ K^−2^ at high temperatures (Figure S8, Supporting Information). Upon Te doping, the *PF* initially increases and then decreases, with a maximum value of 2.1 µW cm^−1^ K^−2^ achieved at 800 K for *x* = 0.167 sample. After further optimization of the carrier concentration, the maximum *PF* is enhanced to 3 µW cm^−1^ K^−2^, which is not as high as compared to that of benchmark TE materials such as Bi_2_Te_3_,^[^ ^41^ ^]^ PbTe,^[^ ^42^ ^]^ and Mg_3_Sb_2_.^[^ ^43^ ^]^ Nevertheless, by leveraging its extremely low *κ_L_*, a promising peak *zT* of 1.0 is attained at 800 K for Ag_8_SnS_5‐_ *_y_* Te samples (Figure 5f). This value represents a fivefold improvement compared to pristine Ag_8_SnS_6_ and is higher than that of most argyrodite‐type compounds.

### 2.5 Lattice Softening Induced by Atomic Mismatch in Other Materials

**Figure 6:**
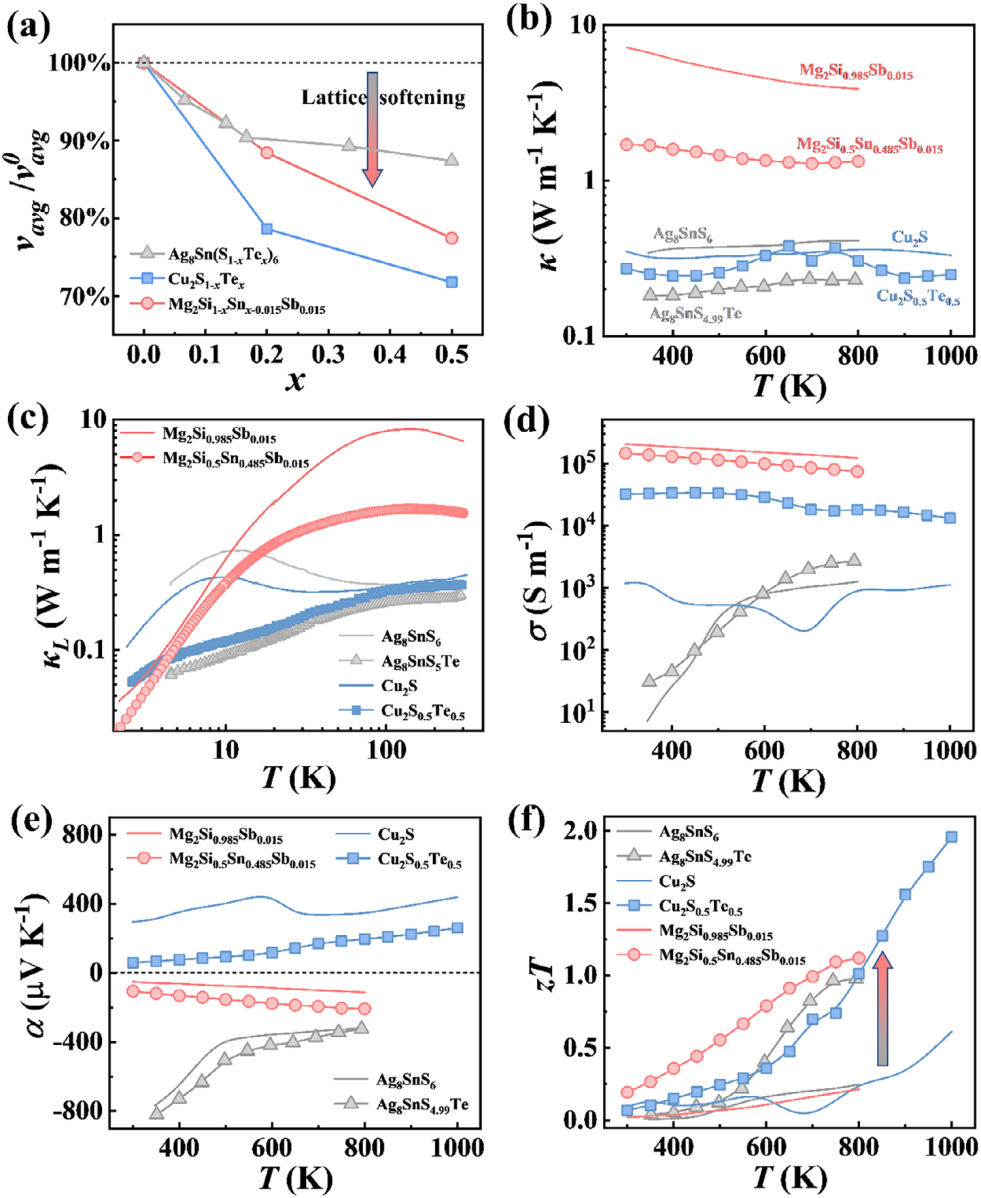
Atomic mismatch‐induced lattice softening leads to high TE performance. a) Average sound velocities (normalized by the value of *x* = 0 sample) plotted against the alloying content *x* for Ag_8_Sn(S_1‐_ *_x_* Te*_x_*)_6_, Cu_2_S_1‐_ *_x_* Te*_x_*, and Mg_2_Si_1‐_ *_x_* Sn*_x‐_*_0.015_Sb_0.015_. The data of Cu_2_S are taken from ref.[44] Temperature dependence of b) total thermal conductivity, c) low temperature lattice thermal conductivity, d) electrical conductivity, e) Seebeck coefficient, and f) TE figure of merit *zT*. The data of Cu_2_S are taken from ref.[44] and the data of Cu_2_S_0.5_Te_0.5_ at 1000 K have been reported in our previous work.^[^ ^31^ ^]^

To demonstrate the general applicability of the lattice softening strategy induced by atomic size mismatch, we extend our studies to another two metaphase systems, i.e., Cu_2_S_1‐_ *_x_* Te*_x_* and Mg_2_Si_0.985‐_ *_x_* Sn*_x_* Sb_0.015_. The PXRD and EDS results (Figures S9 and S10, Supporting Information) indicate that all samples are pure phases despite the significant atomic mismatch between Te and S or between Si and Sn (Table S1, Supporting Information). With increasing Te or Sn content, the transverse, longitudinal, and average sound velocities all decrease sharply (**Figure** **6**a; Table S7, Supporting Information). The average velocity *v_avg_* of Cu_2_S_0.5_Te_0.5_ is only 1593 m s^−1^, which represents a 28% reduction compared to pristine Cu_2_S (2220 m s^−1^). The *v_avg_* of Mg_2_Si_0.5_Sn_0.485_Sb_0.015_ is also much lower than that of the Mg_2_Si matrix. We believe that the reduction in sound velocity of Cu_2_S_1‐_ *_x_* Te*_x_* and Mg_2_Si_0.985‐_ *_x_* Sn*_x_* Sb_0.015_ is also due to the softening of chemical bonds and the increase in average atomic mass.

Similarly, in the Cu_2_S_1‐_ *_x_* Te*_x_* and Mg_2_Si_0.985‐_ *_x_* Sn*_x_* Sb_0.015_ systems, we also observe a marked reduction in the lattice thermal conductivity *κ*_L_ (Figure 6b). Due to the strong liquid‐like behavior,^[^ ^14^, ^44^ ^]^ the *κ*_L_ of Cu_2_S matrix is already as low as 0.35 W m^−1^ K^−1^. Although the introduction of highly mismatched Te atoms may somewhat weaken this effect,^[^ ^45^, ^46^ ^]^ the *κ*_L_ of Cu_2_S_1‐_ *_x_* Te*_x_* is further reduced to 0.27 W m^−1^ K^−1^ because of the significant decrease in sound velocity. Pristine Mg_2_Si_0.985_Sb_0.015_ has a high *κ*_L_ up to 7.2 W m^−1^ K^−1^ at room temperature. Alloying Sn at the Si sites could not only lower the sound velocity but also scatter phonons to reduce the phonon relaxation time. Consequently, the room temperature *κ*_L_ of MgSi_0.485_Sn_0.5_Sb_0.015_ is decreased to ≈1.7 W m^−1^ K^−1^, a threefold reduction compared to that of Mg_2_Si_0.985_Sb_0.015_. Low‐temperature thermal conductivity measurements further show that Cu_2_S_1‐_ *_x_* Te*_x_* and Mg_2_Si_0.985‐_ *_x_* Sn*_x_* Sb_0.015_ metaphases exhibit amorphous thermal transport behavior after introducing mismatched atoms into the structure (Figure 6c).

Cu_2_S_1‐_ *_x_* Te*_x_* is a p‐type semiconductor, while Mg_2_Si_0.985‐_ *_x_* Sn*_x_* Sb_0.015_ is an n‐type semiconductor. For Cu_2_S_1‐_ *_x_* Te*_x_*, the weakening of Cu─X bonds leads to an increase in Cu vacancies.^[^ ^31^ ^]^ This promotes a higher hole concentration, which in turn results in a decrease in the Seebeck coefficient and an increase in electrical conductivity (Figure 6d,e). In the case of Mg_2_Si_0.985‐_ *_x_* Sn*_x_* Sb_0.015_, the weakening of Mg─Y (Y = Si, Sn, Sb) bonds leads to a reduction in electron concentrations, thereby decreasing the electrical conductivity and increasing the Seebeck coefficient (Figure 6d,e). Consequently, the peak *zT* values are boosted to 2.0 for Cu_2_S_0.5_Te_0.5_ and 1.1 for Mg_2_Si_0.5_Sn_0.485_Sb_0.015_, representing a two‐fold and three‐fold increase compared to pristine Cu_2_S and Mg_2_Si_0.985_Sb_0.015_, respectively (Figure 6f).

## 3 Conclusion

In summary, this work demonstrates the validity and universality of atomic mismatch‐induced lattice softening in three metaphase systems: Ag_8_Sn(S,Te), Cu_2_(S,Te) and Mg_2_(Si,Sn). Substituting Te for S or Sn for Si increases the atomic mass and weakens the chemical bonds, leading to a substantial reduction in sound velocities. As a result, these materials exhibit exceptionally low, glass‐like thermal conductivities, reaching as low as 0.15 W m^−1^ K^−1^ at room temperature. This approach is particularly effective for materials with intrinsically low thermal conductivity, as demonstrated in Ag_8_Sn(S,Te) and Cu_2_(S,Te). Ultimately, we achieve excellent thermoelectric performance in these metaphase systems. Unlike strategies based on superionic conduction or other specific structural characters, the lattice‐softening approach proposed here arises from a generalizable design principle: introducing atomic mismatch to weaken bonds and reduce phonon velocities. Beyond thermoelectrics, our approach holds promise for controlling thermal conductivity in applications such as thermal insulation coatings, thermal interface materials, and cryogenics.

## 4 Experimental Section

### Synthesis

A series of Te alloyed Ag_8_Sn(S_1‐_ *_x_* Te*_x_*)_6_ (*x* = 0, 0.067, 0.133, 0.167, 0.333, 0.5, 0.583), S deficient Ag_8_SnS_5‐_ *_y_* Te (*y* = 0.01, 0.02, 0.03), and Te alloyed Cu_2_S_1‐_ *_x_* Te*_x_* (*x* = 0.2, 0.5) samples were synthesized by the melting‐annealing technique, while Sn alloyed Mg_2_Si_1‐_ *_x_* Sn*_x‐_*_0.015_Sb_0.015_ (*x* = 0.015, 0.2, 0.5) samples were synthesized through the high‐energy ball milling technique. For Ag_8_Sn(S_1‐_ *_x_* Te*_x_*)_6_ and Ag_8_SnS_5‐_ *_y_* Te, the mixtures of elements Ag, Sn, S, and Te with high purity (99.999%, Alfa Aesar) were weighed out in stoichiometric proportions and loaded into the silica vacuum tubes for melting. The melting is conducted at 1373 K for 12 h under vacuum, followed by annealing at 823 K for 72 h. After cooling down to room temperature, the ingots were ground into fine powders and then sintered using a hot press system in a graphite die at a peak temperature of ≈973 K and a pressure of 50 MPa for 2 h. For Cu_2_S_1‐_ *_x_* Te*_x_*, the admixture was loaded into a Boron Nitride crucible sealed in an evacuated fused silica tube. The loaded tube was slowly heated to 1423 K in 24 h and dwelt for 12 h before quenched in water to room temperature. Then, the loaded tube was heated to 923 K in 8 h, dwelt for 6 days, and furnace‐cooled to room temperature. The obtained Cu_2_S_1‐_ *_x_* Te*_x_* ingots were crushed and ground to fine powders using a mortar and pestle. The powders were loaded into a graphite die with an inner diameter of 10 mm and consolidated by spark plasma sintering (Sumitomo® SPS‐2040) at 873 K under a pressure of 60 MPa for 5 min. For Mg_2_Si_1‐_ *_x_* Sn*_x‐_*_0.015_Sb_0.015_, high‐purity magnesium (99.5%, Aladdin), silicon (99.99%, Aladdin), tin (99.99%, Aladdin), and antimony (99.99%, Aladdin) powders were weighed out and then loaded into the stainless‐steel ball milling jars in a glove box. It is noted that 8 mol% excess magnesium powder was added to compensate the loss of magnesium during the synthesis. Subsequently, the powders were mixed up for 30 min using high‐energy ball milling machine MSK‐SFM‐3‐1 with a rotate speed of 400 r min^−1^, followed by ball‐milling for 20–40 h in a high‐energy ball milling machine MSK‐SFM‐3‐1 with a rotate speed of 1200 r min^−1^. The obtained powders were then loaded into a graphite die with an inner diameter of 10 mm and pressed into bulk samples by spark plasma sintering (SPS) at 900 K or 1000 K for 15 min under a pressure of 80 MPa. The relative densities of all the obtained bulk samples were larger than 99%. The bulk samples were cut into rectangular (≈2 × 2 × 9 mm^3^) and disks (ϕ10 × 1 mm^2^) for electrical and thermal conductivity measurements, respectively.

### Characterization

To identify the phase purity and crystal structure, the powder X‐ray diffraction (PXRD) data for all synthesized samples were collected on a powder X‐ray diffractometer (D8 Advance) with a Cu K_α_ radiation (*λ* = 1.5406 Å). The Rietveld refinement was performed using the program Jana2006.^[^ ^47^ ^]^ Phase composition and microstructure analysis were carried out by the scanning electron microscopy (SEM, ZEISS Supra 55) equipped with an energy dispersive spectrometer (EDS, Oxford Horiba 250). The heat flow curves of four Ag_8_Sn(S_1‐_ *_x_* Te*_x_*)_6_ (*x* = 0, 0.167, 0.333, 0.5) samples were measured in the temperature range of 200–550 K by a differential scanning calorimeter (DSC, Netzsch 200 F3). The electrical transport properties (*σ* and *S*) from 350 K to 850 K were measured using a ULVAC‐RIKO ZEM‐3 instrument system. The thermal diffusivity (*D*) was measured by the laser flash method using Netzsch LFA 457 instrument, and all the samples were precoated with graphite spray before the measurement. The Dulong–Petit rule was used to estimate the heat capacities (*C_p_*) above 350 K. The sample density *d* was measured using Archimedes' method. The thermal conductivity above 350 K was then calculated based on the equation *κ* = *dDC_p_*. The transverse and longitudinal sound velocities at room temperature were obtained by using the ultrasonic pulse method on the Advanced Ultrasonic Measurement System (UMS, TECLAB). The average sound velocity *v_avg_* is calculated as

(3) $$v_{avg}={\left[\frac{1}{3}\left(\frac{1}{v_{l}^{3}}+\frac{2}{v_{t}^{3}}\right)\right]}^{-1/3}$$

The thermal conductivity in the low temperature range (2–300 K) was measured by a Physical Property Measurement System (PPMS, Quantum Design).

### Theoretical Calculations

First‐principles calculations were carried out with the Vienna Ab initio Simulation Package (VASP) code by utilizing the projector augmented wave (PAW) method for the interaction between ion cores and valence electrons. The low‐temperature ordered structures of Ag_8_SnS_6_ and Ag_8_SnS_5_Te were used for calculation. Both the atomic coordinates and lattice vectors were fully relaxed until the force‐convergence criterion reached 0.01 eV Å^−1^ for Ag_8_SnS_6_ and Ag_8_SnS_5_Te structures. The Perdew Burke Ernzerhof (PBE) functional within Generalized gradient approximation (GGA) was used for treating the electronic exchange and correlation potential. The plane wave energy cutoff was set to 400 eV for all calculations. The crystal orbital Hamilton populations (COHP) were calculated through LOBSTER code^[^ ^48^ ^]^ based on a ground‐state self‐consistent calculation from VASP. The Brillouin zone was sampled by 3 × 3 × 3 Γ‐centered *k* mesh for structural relaxations and charge density calculations, and by 9 × 9 × 9 Γ‐centered *k* mesh for COHP calculations. A rotationally invariant Hubbard *U* correction was adopted to describe the on‐site Coulomb interactions among Ag 4*d* orbital electrons (*U* = 2.0 eV). The Bader charge was analyzed by the Bader code from Henkelman's research group. Phonon calculations were performed using the finite‐displacement method in Phonopy on the relaxed structures, which were optimized with high precision using the SCAN functional.^[^ ^49^ ^]^ The *E_vac_* of an Ag vacancy (*V*_Ag_) was calculated according to the equation

(4) $$E_{vac}=E_{\mathrm{tot},V_{Ag}}+E_{Ag}-E_{\mathrm{tot}}$$

where *E*_tot_ and $E_{\mathrm{tot},V_{Ag}}$ are the cohesive energies of the supercell before and after the introduction of an Ag vacancy, respectively; *E_Ag_* is the cohesive energy of silver with fcc crystal structure.

## Conflict of Interest

The authors declare no conflict of interest.

## Supporting information



Supporting Information

## Data Availability

The data that support the findings of this study are available from the corresponding author upon reasonable request.
