# Supplementary material for: Strong Lattice Softening Induced by Atomic Mismatch in Meta‐Phase Thermoelectrics
Source: Adv Sci (Weinh). 2025 Oct 6;12(48):e14105. doi: 10.1002/advs.202514105 (PMC12752634; doi:10.1002/advs.202514105)
Supplement: Supplementary file 1 — Supporting Information [file ADVS-12-e14105-s001.docx]

**Supplementary Information**

Strong Lattice Softening Induced by Atomic Mismatch in Meta-phase Thermoelectrics

*Kunpeng Zhao^1,#,*^, Min Li^2,3,4,#^, Hexige Wuliji^1^, Haotian Gao^1^, Hongyi Chen^5^, Pengfei Qiu^2^, Zhengyang Zhou^2*^, and Xun Shi^2,3*^*

^#^ These authors contributed equally

^*^Corresponding author. Email: [zkp.1989@sjtu.edu.cn](mailto:zkp.1989@sjtu.edu.cn); [zhouzhengyang@mail.sic.ac.cn](mailto:zhouzhengyang@mail.sic.ac.cn); [xshi@mail.sic.ac.cn](mailto:xshi@mail.sic.ac.cn)


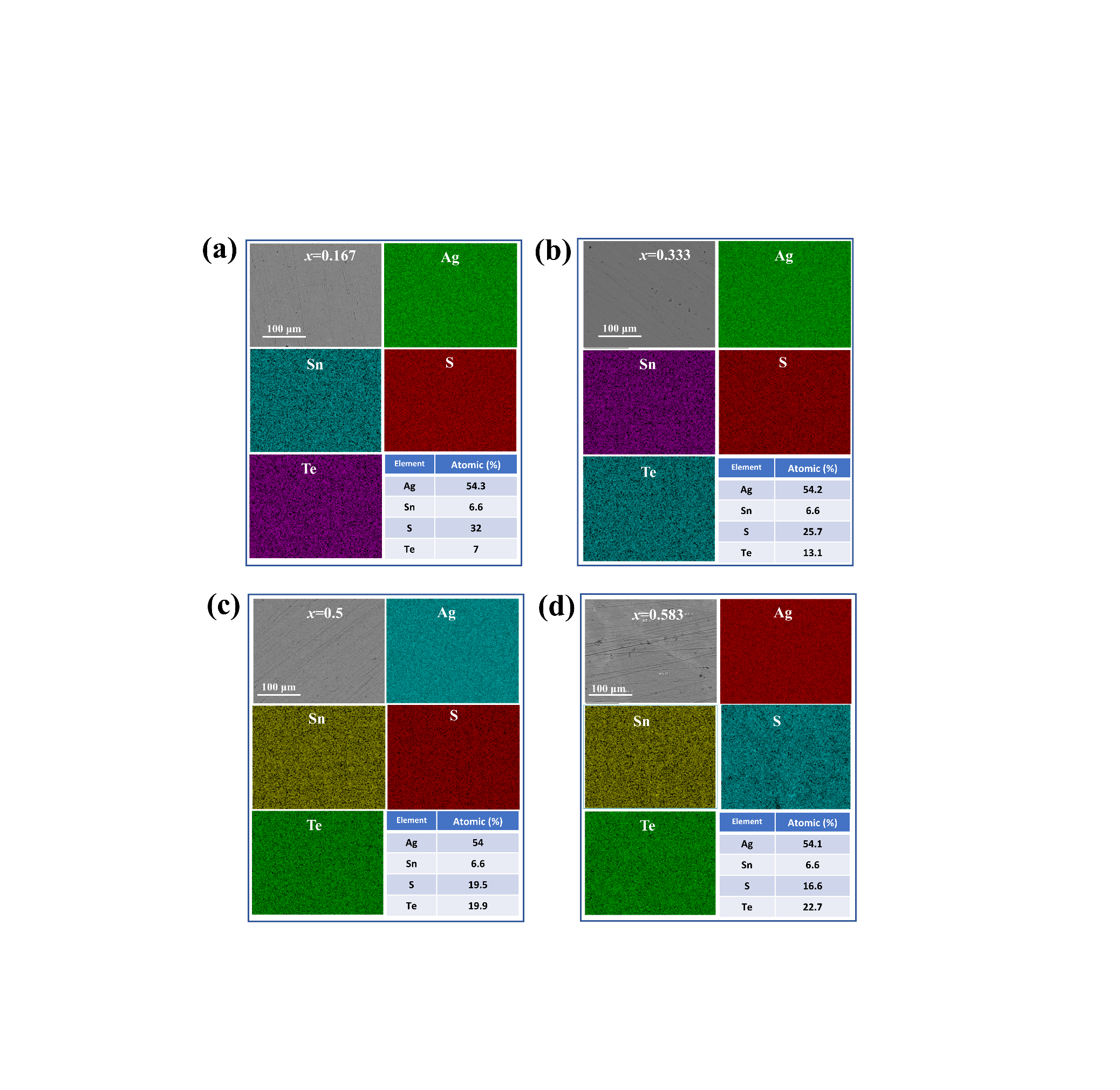


**Fig. S1**. Elemental energy-dispersive X-ray spectroscopy (EDS) mapping of Ag_8_Sn(S_1-_*_x_*Te*_x_*)_6_ (*x* = 0.167, 0.333, 0.5, 0.583) samples.


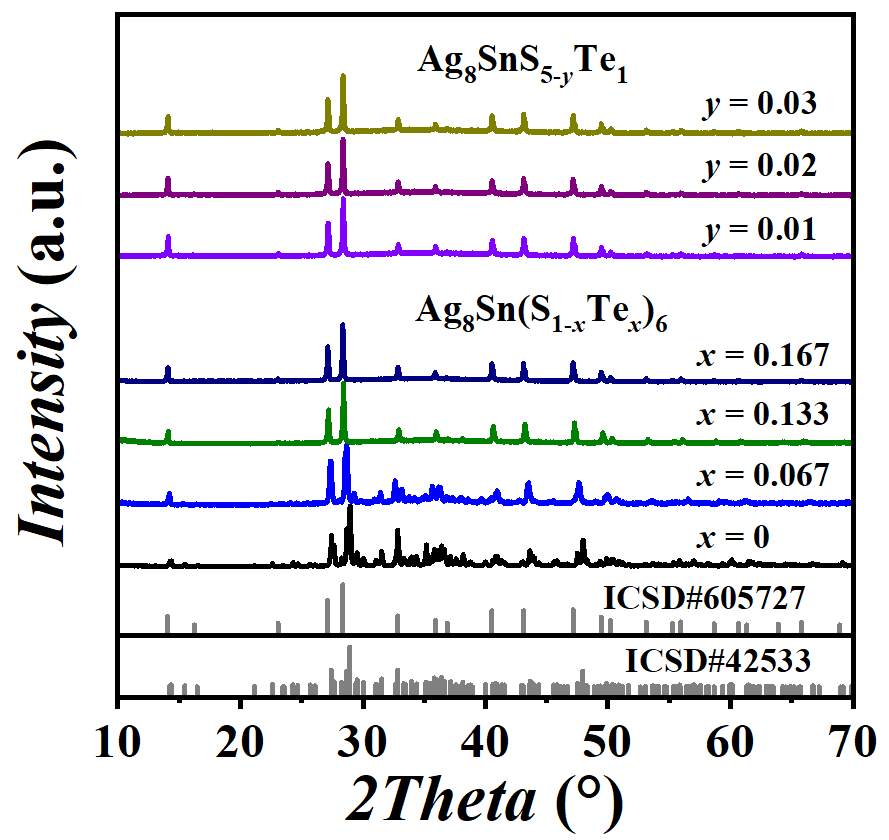


**Fig. S2**. Room temperature powder X-ray diffraction (XRD) patterns for Ag_8_Sn(S_1-_*_x_*Te*_x_*)_6_ (*x* = 0, 0.067, 0.133, 0.167) and Ag_8_SnS_5-_*_y_*Te (*y* = 0.01, 0.02, 0.03) samples.

**Fig. S3.** High temperature X-ray diffraction patterns for Ag_8_Sn(S_1-_*_x_*Te*_x_*)_6_ (*x* = 0, 0.167, 0.333).

**
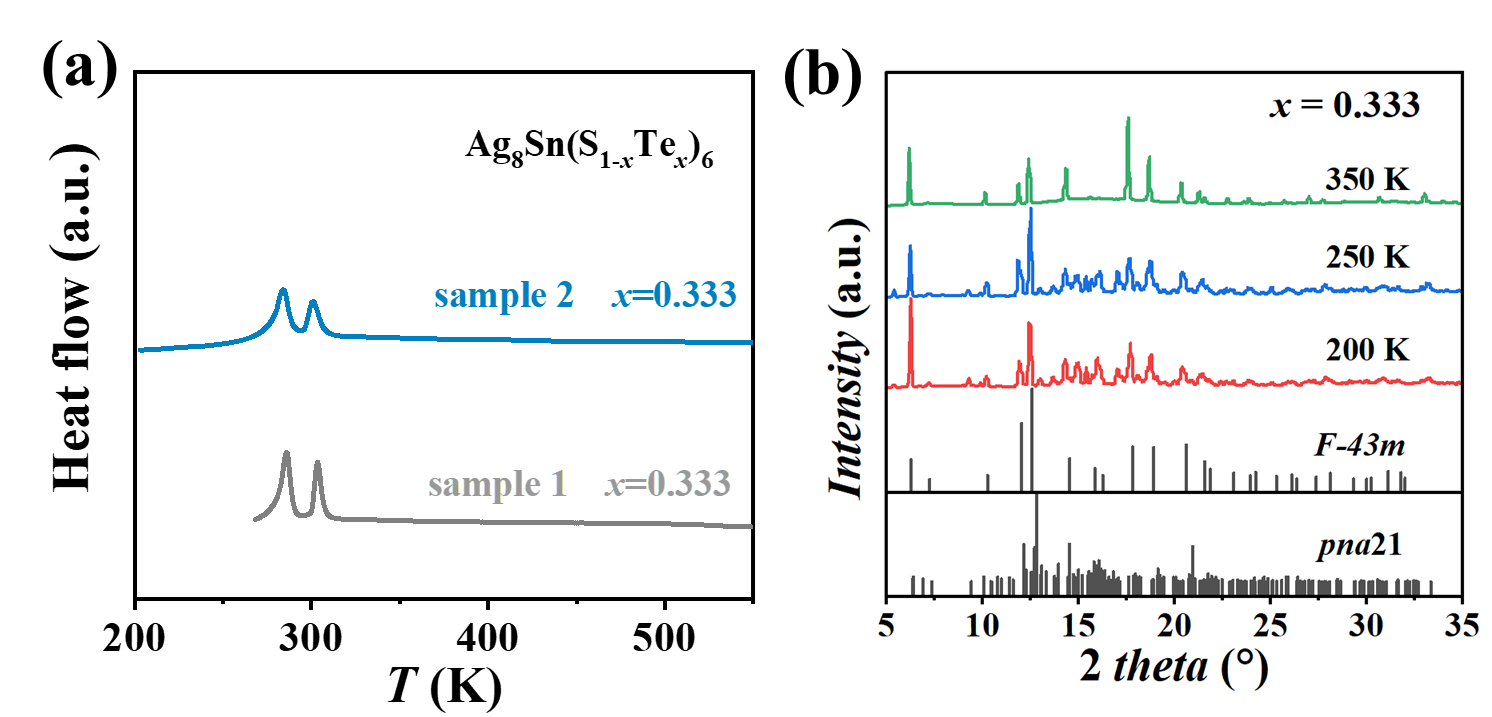
**

**Fig. S4**. **(a)** Temperature dependence of heat flow for two independent *x* = 0.333 sample. **(b)** Variable-temperature XRD patterns for *x* = 0.333 sample.


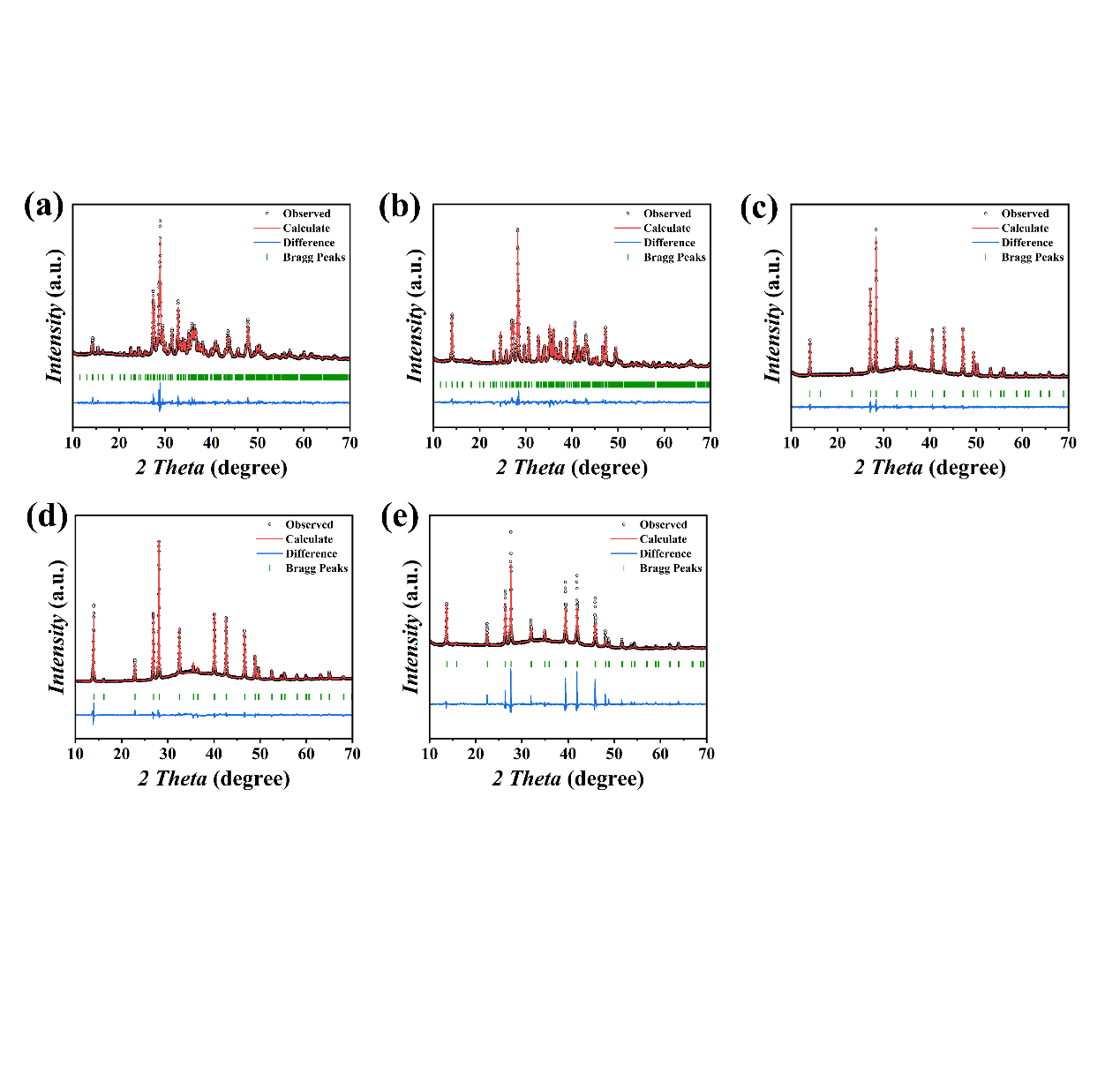


**Fig. S5**. Rietveld reﬁnements for Ag_8_SnS_6-_*_x_*Te*_x_* samples and the overlaid calculated pattern (dashed line) with diﬀerence proﬁle below. **(a)** orthorhombic Ag_8_SnS_6_, **(b)** orthorhombic Ag_8_SnS_5_Te, **(c)** cubic Ag_8_SnS_5_Te, **(d)** cubic Ag_8_SnS_4_Te_2_, and **(e)** cubic Ag_8_SnS_3_Te_3_.


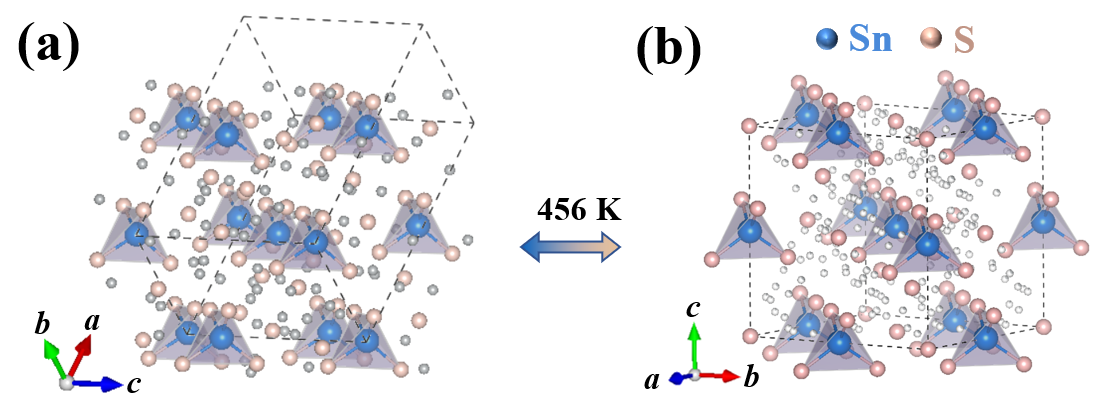


**Fig. S6**. Phase transition from the **(a)** low-temperature orthorhombic structure to **(b)** the high-temperature cubic structure for Ag_8_SnS_6_.


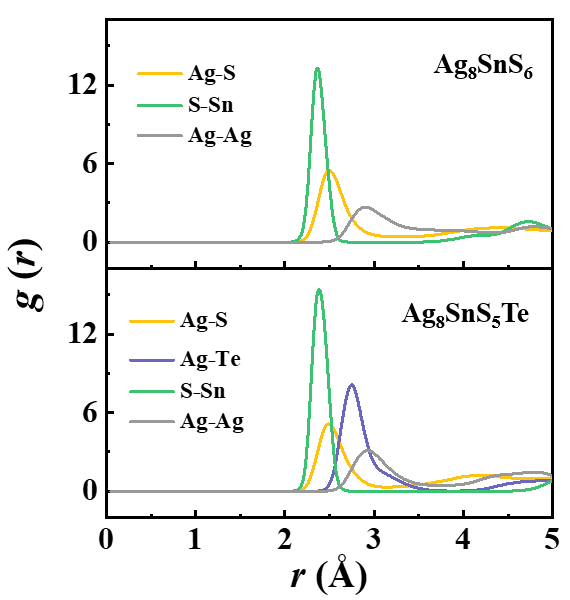


**Fig. S7**. Radial distribution functions (RDF) of Ag_8_SnS_6_ and Ag_8_SnS_5_Te calculated from first-principles molecular dynamics.


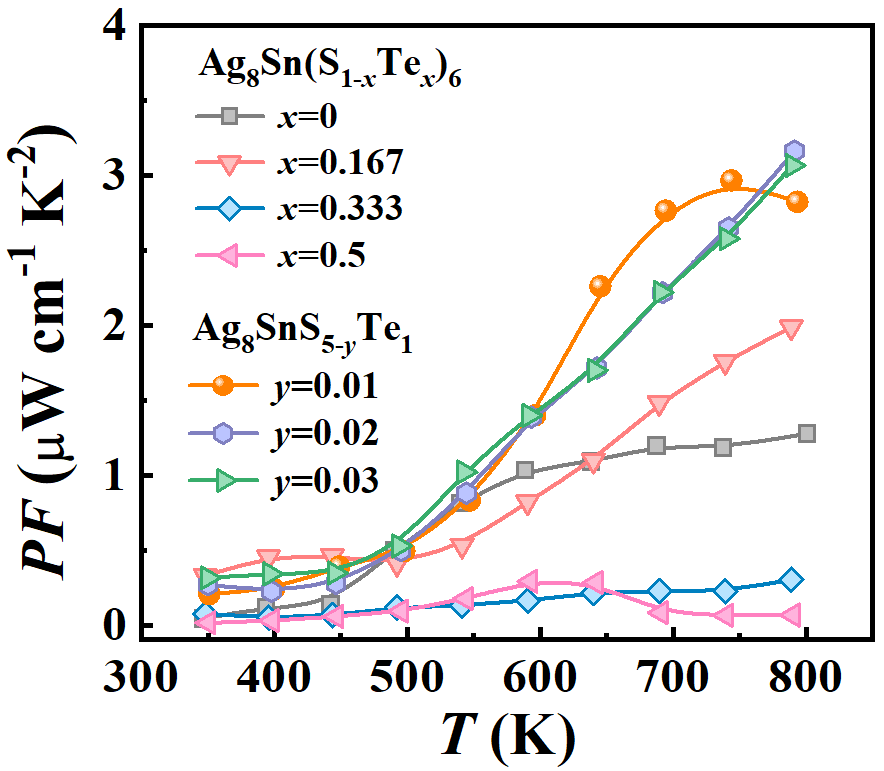


**Fig. S8**. Temperature dependence of power factor *PF* for Ag_8_Sn(S_1-_*_x_*Te*_x_*)_6_ and Ag_8_SnS_5-_*_y_*Te.


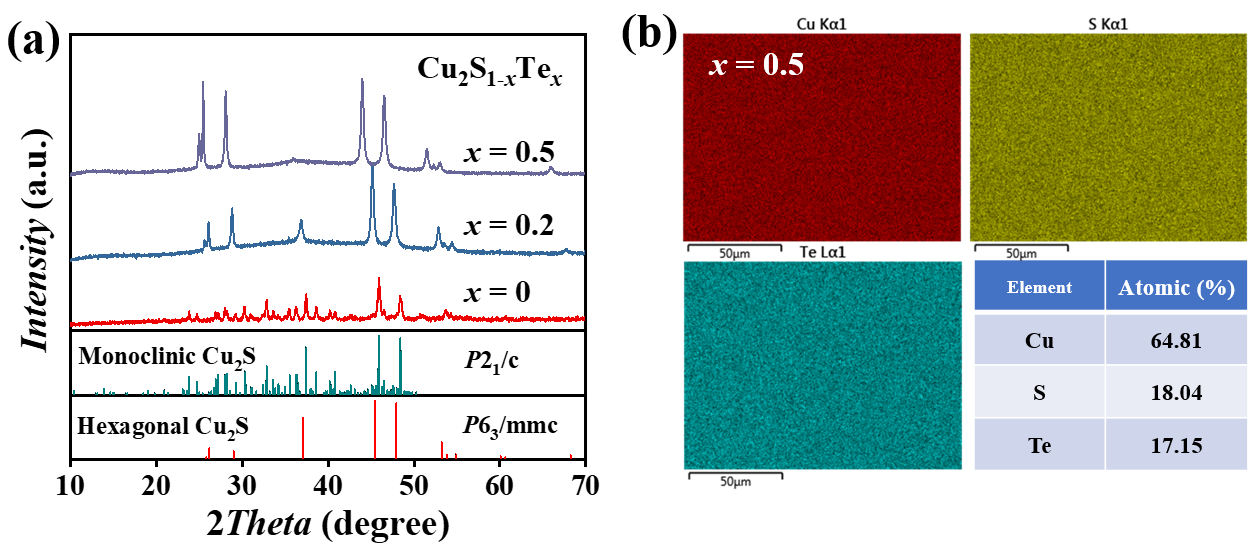


**Fig. S9**. Room temperature PXRD patterns for Cu_2_S_1-_*_x_*Te*_x_* (*x* = 0, 0.2, 0.5) and EDS mapping for *x* = 0.5 sample. The XRD results have been reported in our previous study ^[1]^. Upon alloying Te, Cu_2_S_1-_*_x_*Te*_x_* undergoes a structural transition from monoclinic phase to hexagonal phase.


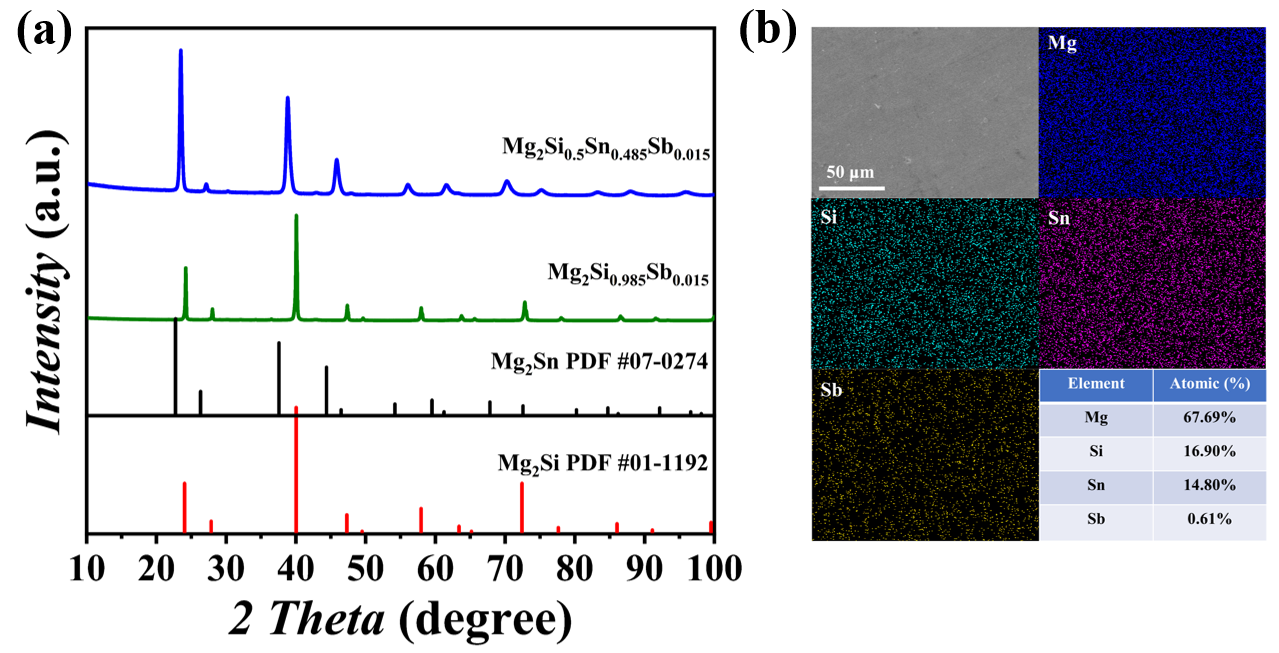


**Fig. S10**. Room temperature PXRD patterns for Mg_2_Si_1-_*_x_*Sn*_x-_*_0.015_Sb_0.015_ (*x* = 0 or 0.5) and EDS mapping for *x* = 0.5 sample.

**Table S1.** Atomic radius, ionic radius, relative atomic mass and electronegativity of elements S, Te, Si, and Sn.

| elements | atomic radius | ionic radius | relative atomic mass | electronegativity |
| --- | --- | --- | --- | --- |
| S | 0.88 Å | 1.84 Å (-2) | 32.1 | 2.58 |
| Te | 1.23 Å | 2.21 Å (-2) | 127.6 | 2.1 |
| Si | 1.11 Å | 0.4 Å (+4) | 28.1 | 1.90 |
| Sn | 1.45 Å | 0.69 Å (+4) | 118.7 | 1.96 |

**Table S2.** Refined atomic parameters solved from powder X-ray diffraction data of the orthorhombic structure of Ag_8_SnS_6_ at room temperature.

| **Atom** | **Occupancy** | ***x*/a** | ***y*/b** | ***z*/c** | **U** |
| --- | --- | --- | --- | --- | --- |
| **Ag1** | 1 | 0.1248(5) | 0.2198(11) | 0.3836(12) | 0.062(4) |
| **Ag2** | 1 | 0.0622(5) | 0.2252(11) | 0.8340(13) | 0.070(3) |
| **Ag3** | 1 | 0.4385(6) | 0.0628(9) | 0.0165(11) | 0.050(3) |
| **Ag4** | 1 | 0.2773(6) | 0.5155(11) | 0.0854(12) | 0.074(4) |
| **Ag5** | 1 | 0.4110(7) | 0.0810(11) | 0.7004(13) | 0.084(4) |
| **Ag6** | 1 | 0.2768(5) | 0.3740(9) | 0.6867(11) | 0.064(3) |
| **Ag7** | 1 | 0.0183(6) | 0.0161(14) | 0.6017(13) | 0.066(4) |
| **Ag8** | 1 | 0.2590(7) | 0.1575(11) | 0.9356(12) | 0.087(4) |
| **Sn1** | 1 | 0.3744(3) | 0.2305(8) | 0.3523 | 0.0140(17) |
| **S1** | 1 | 0.1274(18) | 0.484(2) | 0.975(2) | 0.000(2) |
| **S2** | 1 | 0.0056(16) | 0.257(4) | 0.2218(17) | 0.000(2) |
| **S3** | 1 | 0.3733(16) | 0.475(2) | 0.490(2) | 0.000(2) |
| **S4** | 1 | 0.2440(16) | 0.239(2) | 0.2096(18) | 0.000(2) |
| **S5** | 1 | 0.3959(11) | 0.321(2) | 0.873(3) | 0.000(2) |
| **S6** | 1 | 0.1201(13) | 0.276(2) | 0.615(2) | 0.000(2) |
| R_p_ = 0.025, wR_p_ = 0.033, R_obs_ = 0.0443, wR_obs_ = 0.0412, R_all_ = 0.0461, wR_all_ = 0.0415 | | | | | |

**Table S3.** Refined atomic parameters solved from powder X-ray diffraction data of the orthorhombic structure of Ag_8_SnS_5_Te at room temperature.

| **Atom** | **Occupancy** | ***x*/a** | ***y*/b** | ***z*/c** | **U** |
| --- | --- | --- | --- | --- | --- |
| **Ag1** | 1 | 0.5485(8) | 0.5563(17) | 0.5341(11) | 0.038(4) |
| **Ag2** | 1 | 0.5485(9) | -0.0354(17) | 0.5574(12) | 0.078(6) |
| **Ag3** | 1 | 0.2321(7) | 0.0266(16) | 0.6206(11) | 0.069(6) |
| **Ag4** | 1 | 0.6364(3) | 0.2476(19) | 0.4139(9) | 0.047(3) |
| **Ag5** | 1 | 0.2469(8) | 0.4524(16) | 0.6431(12) | 0.083(6) |
| **Ag6** | 1 | 0.4262(9) | 0.444(2) | 0.7449(15) | 0.089(6) |
| **Ag7** | 1 | 0.3036(5) | -0.244(2) | 0.3805(13) | 0.101(3) |
| **Ag8** | 1 | 0.5813(9) | -0.043(2) | 0.2524(15) | 0.087(7) |
| **Sn1** | 1 | 0.3784(2) | 0.2494(16) | 0.383137 | 0.0113(15) |
| **S1** | 0.3 | 0.0938(3) | 0.249(2) | 0.6609(8) | 0.022(2) |
| **S2** | 0.7 | 0.3521(5) | 0.217(2) | 0.8974(18) | 0.029(4) |
| **S3** | 1 | 0.3753(17) | 0.021(4) | 0.541(2) | 0.009(3) |
| **S4** | 1 | 0.5060(12) | 0.259(7) | 0.2560(16) | 0.009(3) |
| **S5** | 1 | 0.2583(12) | -0.228(5) | 0.7663(17) | 0.009(3) |
| **S6** | 1 | 0.3844(18) | 0.502(3) | 0.500(2) | 0.009(3) |
| **Te1** | 0.7 | 0.0938(3) | 0.249(2) | 0.6609(8) | 0.022(2) |
| **Te2** | 0.3 | 0.3521(5) | 0.217(2) | 0.8974(18) | 0.029(4) |
| R_p_ = 0.0265, wR_p_ = 0.033, R_obs_ = 0.0535, wR_obs_ = 0.0502, R_all_ = 0.0567, wR_all_ = 0.0507 | | | | | |

**Table S4.** Refined atomic parameters solved from powder X-ray diffraction data of the cubic structure of Ag_8_SnS_5_Te at room temperature.

| **Atom** | **Occupancy** | ***x*/a** | ***y*/b** | ***z*/c** | **U** |
| --- | --- | --- | --- | --- | --- |
| **Ag1** | 0.074(5) | -0.021(2) | 0.090(2) | 0.1746(13) | 0.028(3) |
| **Ag2** | 0.210(9) | 0.1635(7) | 0.1635(7) | 0.0481(19) | 0.028(3) |
| **Ag3** | 0.309(6) | 0.2099(6) | 0.2099(6) | -0.0138(4) | 0.028(3) |
| **Sn1** | 1 | 0.50000 | 0.50000 | 0.50000 | 0.0578(17) |
| **S1** | 1 | 0.6287(7) | 0.6287(7) | 0.6287(7) | 0.001 |
| **S2** | 0.700 | 0.00000 | 0.00000 | 0.00000 | 0.045(3) |
| **S3** | 0.300 | 0.25000 | 0.25000 | 0.25000 | 0.065(3) |
| **Te1** | 0.300 | 0.00000 | 0.00000 | 0.00000 | 0.045(3) |
| **Te2** | 0.700 | 0.25000 | 0.25000 | 0.25000 | 0.065(3) |
| R_p_ = 0.0249, wR_p_ = 0.0323, R_obs_ = 0.0325, wR_obs_ = 0.0383, R_all_ = 0.0376, wR_all_ = 0.0384 | | | | | |

**Table S5.** Refined atomic parameters solved from powder X-ray diffraction data of the cubic structure of Ag_8_SnS_4_Te_2_ at room temperature.

| **Atom** | **Occupancy** | ***x*/a** | ***y*/b** | ***z*/c** | **U** |
| --- | --- | --- | --- | --- | --- |
| **Ag1** | 0.095(7) | 0.0688(6) | 0.0688(6) | 0.1858(12) | 0.0717(14) |
| **Ag2** | 0.287(5) | 0.1652(6) | 0.1652(6) | -0.0103(5) | 0.0717(14) |
| **Ag3** | 0.285(5) | 0.2828(6) | 0.2828(6) | 0.0192(5) | 0.0717(14) |
| **Sn1** | 1 | 0.50000 | 0.50000 | 0.50000 | 0.0279(10) |
| **S1** | 0.975 | 0.6237(3) | 0.6237(3) | 0.6237(3) | 0.0026(13) |
| **S2** | 0.1 | 0.00000 | 0.00000 | 0.00000 | 0.1327(19) |
| **Te1** | 0.025 | 0.6237(3) | 0.6237(3) | 0.6237(3) | 0.0026(13) |
| **Te2** | 0.9 | 0.00000 | 0.00000 | 0.00000 | 0.1327(19) |
| **Te3** | 1 | 0.25000 | 0.25000 | 0.25000 | 0.0736(14) |
| R_p_ = 0.0429, wR_p_ = 0.0630, R_obs_ = 0.0451, wR_obs_ = 0.0542, R_all_ = 0.0482, wR_all_ = 0.0543 | | | | | |

**Table S6.** Refined atomic parameters solved from powder X-ray diffraction data of the cubic structure of Ag_8_SnS_3_Te_3_ at room temperature.

| **Atom** | **Occupancy** | ***x*/a** | ***y*/b** | ***z*/c** | **U** |
| --- | --- | --- | --- | --- | --- |
| **Ag1** | 0.1596 | 0.0703(12) | 0.0703(12) | 0.199(2) | 0.096(5) |
| **Ag2** | 0.351 | 0.1768(9) | 0.1768(9) | -0.0082(10) | 0.096(5) |
| **Ag3** | 0.156 | 0.272(2) | 0.272(2) | 0.024(2) | 0.096(5) |
| **Sn1** | 1 | 0.50000 | 0.50000 | 0.50000 | 0.053(4) |
| **S1** | 0.75 | 0.6291(6) | 0.6291(6) | 0.6291(6) | 0.021(3) |
| **Te1** | 0.25 | 0.6291(6) | 0.6291(6) | 0.6291(6) | 0.021(3) |
| **Te2** | 1 | 0.00000 | 0.00000 | 0.00000 | 0.131(6) |
| **Te3** | 1 | 0.25000 | 0.25000 | 0.25000 | 0.073(5) |
| R_p_ = 0.0403, wR_p_ = 0.0698, R_obs_ = 0.0412, wR_obs_ = 0.0383, R_all_ = 0.0462, wR_all_ = 0.0388 | | | | | |

**Table S7.** Transverse (*v_t_*), longitudinal (*v_l_*) and averaged sound velocity (*v_avg_*) for Ag_8_Sn(S_1-_*_x_*Te*_x_*)_6_, Cu_2_S_1-_*_x_*Te*_x_*, and Mg_2_Si_1-_*_x_*Sn*_x-_*_0.015_Sb_0.015_. The data of Cu_2_S are taken from ref. ^[2]^

| **Samples** | ***ν*_t_**  **(m s^-1^)** | ***ν*_l_**  **(m s^-1^)** | ***v*_avg_**  **(m s^-1^)** |
| --- | --- | --- | --- |
| Ag_8_SnS_6_ | 1400 | 3200 | 1581 |
| Ag_8_SnS_5.6_Te_0.4_ | 1332 | 3107 | 1505 |
| Ag_8_SnS_5.2_Te_0.8_ | 1290 | 3030 | 1458 |
| Ag_8_SnS_5_Te | 1267 | 2828 | 1429 |
| Ag_8_SnS_4_Te_2_ | 1253 | 2702 | 1411 |
| Ag_8_SnS_3_Te_3_ | 1230 | 2509 | 1381 |
| Cu_2_S | 3634 | 1991 | 2220 |
| Cu_2_S_0.8_Te_0.2_ | 3343 | 1550 | 1746 |
| Cu_2_S_0.5_Te_0.5_ | 2835 | 1420 | 1593 |
| Mg_2_Si_0.985_Sb_0.015_ | 7607 | 4670 | 5154 |
| Mg_2_Si_0.8_Sn_0.185_Sb_0.015_ | 6713 | 4131 | 4558 |
| Mg_2_Si_0.5_Sn_0.485_Sb_0.015_ | 6038 | 3605 | 3991 |

**References**

[1] K. Zhao, C. Zhu, W. Qiu, S. Yang, H. Su, P. Qiu, Y. He, M. Guan, T.-R. Wei, J. Ma, Matter 2022, 5, 605.

[2] Y. He, T. Day, T. Zhang, H. Liu, X. Shi, L. Chen, G. J. Snyder, Adv. Mater. 2014, 26, 3974.
